# Effectiveness of Canine Hip Dysplasia and Elbow Dysplasia Improvement Programs in Six UK Pedigree Breeds

**DOI:** 10.3389/fvets.2019.00490

**Published:** 2020-01-15

**Authors:** H. K. James, F. McDonnell, Thomas W. Lewis

**Affiliations:** ^1^The Kennel Club, London, United Kingdom; ^2^School of Veterinary Medicine and Science, The University of Nottingham, Nottingham, United Kingdom

**Keywords:** hip dysplasia, elbow dysplasia, canine health, selection, phenotypic improvement

## Abstract

Hip and elbow dysplasia are common disorders in larger dog breeds and crosses, and a known contributory factor to osteoarthritis, lameness and reduced mobility. Screening schemes evaluating the severity of hip and elbow dysplasia in the UK are administered by the British Veterinary Association (BVA) and the Kennel Club (KC). The BVA/KC Hip Dysplasia scoring scheme is over 50 years old, having originated in 1965, and has operated in its current form since 1983. The BVA/KC Elbow Dysplasia grading scheme commenced more recently in 1998 and is based on the International Elbow Working Group guidelines. Hip score and elbow grade data on a considerable number of dogs in the UK have been generated from these two screening schemes. This study analyses data from dogs of six breeds scored from 1990 to present, to establish any determinable trends in hip score and elbow grade parameters, and to examine whether the implementation of such schemes has had a positive influence on hip and elbow health. A range of criteria, including the rate of participation in the screening schemes, hip score and elbow grade parameters (e.g., median, mean, standard deviation), and estimated breeding values (EBVs) were analyzed, both in the overall population and also among breeding animals. The results show a general decline in hip score parameters (median, mean, standard deviation, and 75th percentile), revealing a reduction in the prevalence and severity of hip dysplasia. There was a more modest decline in mean elbow grade within breeds. The proportion of sires and dams (of dogs born per year) with no hip score or elbow grade fell substantially over time, demonstrating good participation in the screening schemes. In most breeds, the scores of sires and dams are demonstrably improving. There is a declining genetic trend as ascertained by EBVs for both hip scores and elbow grades in most breeds, implying that the improvement observed is due in part to selection for improvement in hip and elbow health as described by the respective screening schemes.

## Introduction

Hip and elbow dysplasia are considered important hereditary orthopedic diseases that are known to be prevalent across several, in particular medium to large, dog breeds and their crosses, and have long been widely acknowledged to negatively impact the health and welfare of an affected individual (1). Hip and elbow dysplasia are categorized as developmental disorders caused by dysmorphic and lax joint formation. This malformation consequentially results in abnormal wearing of bone over time, inducing the secondary development of osteoarthritis (OA) or arthrosis, and degenerative joint disease (DJD) (2). Elbow dysplasia can be categorized into four subsets of disease: osteoschondrosis of the medial part of the humeral condyle, fragmented medial coronoid process (FCP), ununited anconeal process (UAP), and incongruity of the elbow joint (3, 4). Unfortunately, the pathology of neither hip nor elbow dysplasia can be reversed and so, for an affected individual, the best outcome is management of the disease through pain medication or replacement surgery, with the latter having additional consequences of cost and an extensive recovery period (5). The underlying etiology of dysplastic disease is complex with a long understood knowledge of a genetic influence (6–8) and multiple environmental factors, such as obesity or over-exercise during growth (9–11). Given genetic influences contribute to an individual's risk of development of both hip and elbow dysplasia, the importance of selecting breeding stock with the aim of reducing genetic risk in future generations is seen as the most useful means to elicit a widespread and permanent reduction in disease (2, 12).

While there is an established underlying complex (i.e., non-Mendelian) genetic influence on dysplastic disease, the consequential lameness and DJD does not usually become clinically apparent until after breeding age. Therefore, screening schemes such as the British Veterinary Association (BVA)/Kennel Club (KC) Hip and Elbow Dysplasia Schemes have been established to give an indication of the severity of pre-clinical affectation, and so provide breeders with the ability to make informed decisions regarding which animals to use for breeding. Due to earlier recognition of hip dysplasia as a serious welfare impairment, the BVA/KC Hip Dysplasia scheme was launched in its current format in 1983, with the Elbow Dysplasia scheme following later in 1998, which follows guidelines provided by the International Elbow Working Group (IEWG). Scoring of an individual involves a ventrodorsal and mediolateral radiograph (for hips and elbows, respectively), before submitting to the BVA for scrutiny by a panel of veterinary experts in orthopedics and radiography. The criteria for hip radiograph scrutiny incorporate nine distinct features on each hip, each scored according to the degree of laxity and/OA with a final score established from the sum of the total for the left and right hip (13). With regard to elbows, the margins between the joint and the bone structures are measured, as well as signs of any primary lesions (an area of damage caused directly by disease) and/or OA (14). The minimum score is 0 and the maximum possible is 3, whereby the highest individual elbow score taken is the overall grade (e.g., a right elbow grade of 0 and a left elbow grade of 1 would be reported as grade 1).

For breeds where a significant proportion of the population has participated in dysplasia screening schemes, numerous studies have determined the heritability of various measures of hip dysplasia and elbow dysplasia, i.e., quantifying the extent of additive genetic variation underlying apparent phenotypic variation, across a variety of breeds and countries (12, 15–25). The moderate magnitude of the various estimates of heritability demonstrate that selection for improvement will be successful. Furthermore, in several countries individual estimates of genetic risk, estimated breeding values (EBVs), for evaluations of hip dysplasia (HD) and elbow dysplasia (ED) are routinely provided on registered pedigree dogs. EBVs provide a more accurate metric for selection than phenotypic measures of HD and ED since non-additive genetic (including environmental) effects are discounted and information on relatives (who share genetics) is taken into account. While several different loci having been identified as associated with disease (2, 26–28), it is not always clear what proportion of the additive genetic variance they comprise and it is likely that only genomic breeding values (gEBVs) will offer an effective “DNA test” for dysplastic disorders (29, 30). However, these will, like EBVs, take the form of a quantification of risk, rather than denoting binary categories of “affected” and “unaffected.”

This study utilized screening data of UK Kennel Club registered dogs from six breeds born from 1990 to 2018 to establish any determinable trends in parameters, and to examine whether the implementation of such schemes has had a beneficial influence on overall hip and elbow health. A range of criteria, including the rate of participation in scoring schemes, score parameters (such as median, mean, standard deviation of scores, and grades), and EBVs were analyzed, both in the overall population and also among breeding animals.

## Methods

### Data

Six breeds with EBVs for hip score and elbow grade from the BVA/KC screening schemes were included in the study: Labrador Retriever (LR), Golden Retriever (GR), German Shepherd Dog (GSD), Rottweiler (ROTT), Bernese Mountain Dog (BMD), and Newfoundland (NEWF). Participation in the BVA/KC hip and elbow screening schemes is voluntary and details of scoring protocols are given by Fluckiger (14) and Gibbs (31), respectively. In brief, radiographs of hips are scored bilaterally on 9 features according to the degree of laxity and/or OA observed. Eight features are scored from 0 to 6, and one feature is scored from 0 to 5, zero indicating an absence of, and higher numbers the severity of, pathology. The maximum score, indicating the most severe pathology, for each hip is 53. Both the individual totals for left and right hip are publically reported, along with the bilateral total score which ranges from 0 (indicating no malformation) to 106 (severe hip dysplasia). The BVA/KC elbow scoring scheme was launched in 1998 based on guidelines of the International Elbow Working Group (IEWG). Elbow radiographs are scored according to the size of detectable primary lesions and severity and extent of OA observed; a score of 0 denotes that the elbow is radiographically normal, (1) that signs of mild OA are visible, (2) that a moderate or a primary lesion is present but with no OA, and (3) that there is severe osteoarthritis or a primary lesion with signs of OA. Only the score of the higher elbow grade is publically reported. Pedigree and phenotypic data for the listed breeds were extracted from the Kennel Club electronic databases on 1st April 2019. The EBVs for hip score and elbow grade are re-calculated regularly four times per year using updated pedigree and phenotypic data and are publically accessible via the Kennel Club website. The calculation of best linear unbiased predictor (BLUP) EBVs is as described by Lewis et al. (22, 25), with genetic parameters estimated using ASREML (32) and the BLUP EBVs calculated using MiXBLUP software (https://www.mixblup.eu/index.html). EBVs were retrieved from files generated in their most recent routine update (April, 2019) and used to examine genetic trends.

### Analysis

For each breed included, the number of registered animals born, the number with a hip score; and the median and mean averages, standard deviation, and 75th percentile of those hip scores, each year from 1990 to 2018 inclusive were calculated. Since dogs are required to be over 1 year (365 days) old to participate in the BVA/KC hip (and elbow) screening scheme, the majority of the dogs born in 2018 will have been too young to participate at the time of data extraction (1st April 2019), and so phenotypic data from individuals born in this year was incomplete. Furthermore, given the developmental nature of the disease, younger dogs are known to have lower scores due to less severe pathology (33). Therefore, there is the potential for bias in the scores of cohorts of dogs which are younger, that is born in recent years; e.g., dogs born in 2017 will have been between 15 and 27 months old at time of data extraction, and so scores from older dogs in this cohort are missing, which may bias the parameters. Over 90% of dogs of these breeds are scored before they are 4 years old, so an attempt to minimize potential bias introduction was made by excluding cohorts of 2017, 2016, and 2015 born dogs (which will all have contained dogs under 4 years old at the time of data extraction, and so susceptible to bias). Thus, although the total data extracted comprised dogs born in years up to and including 2018, the score parameters described above of individual 2015–2018 born dogs were excluded from analysis. Therefore, the dataset of hip score parameters on individual registered dogs per year of birth consisted of those dogs born from 1990 to 2014. However, because EBVs are calculated for all animals in the pedigree, including those without phenotypes, analyses were performed on EBV data on dogs born from 1990 to 2018 (the last complete calendar year). EBVs are centered and scaled according to breed-specific parameters from the previous 10 years to give a mean of zero and a standard deviation of ±20, with negative numbers indicating lower genetic risk than 10 year average in the breed.

Finally, per year of birth, the sires and dams of registered animals born were identified, and the proportion of each (sires and dams) with a hip score determined, and the median and mean averages, standard deviation, and 75th percentile of those hip scores calculated. Again, data comprised sires and dams of dogs born from 1990 to 2018.

General analyses of elbow grades included data on individual dogs from the six breeds described born each year from 1998 to 2014 for the same reasons outlined above. The parameters calculated included the proportion of graded dogs, the proportion of total elbow grades (left + right elbow grades) that were zero, and the median, mean and standard deviation of total elbow grade. EBV data on individuals born 1990–2018, and the proportion of sires and dams (of registered dogs born 1990–2018) with elbow grades, and the proportion of total elbow grades equal to zero, were analyzed.

Three year rolling means of parameters over latter years were calculated to provide most recent observed levels for across breed comparison. Linear regression of each parameter of hip score or elbow grade (e.g., mean hip score) on individual year of birth were performed using Matlab (34), and the coefficients (trends) and statistical significance reported along, in some cases, with the R-squared value, which is the proportion of variation in the dependent variable explained by the progressing year of birth.

## Results

### Hips

#### Individual Score Parameters Over Year of Birth

There was variation across the six breeds in the proportion of registered animals born per year that had undergone hip screening and so had hip scores, the 3 year rolling mean proportion over 2012–2014 being 7.83% in LR, 10.62% in GR, 8.39% in GSD, 10.05% in ROTT, 18.80% in BMD, and 13.98% in NEWF. Results from linear regression of the percentage of registered animals born that have undergone screening on individual year of birth from 1990 to 2014 revealed varied coefficients; there were statistically significant negative trends in three breeds, LR (−0.0796%), BMD (−0.2546%), and NEWF (−0.3996%) and statistically significant positive trends in the GR (+0.0948%) and GSD (+0.0732%), with no significant trend in ROTT. However, none of the detected regression coefficients were large in magnitude (Table 1). Raw data on the proportion of registered animals born per year with hip scores are given in individual breed tables in Supplementary Table 1.

**Table 1:** Regression coefficients (describing trend) of hip score parameters listed on year of birth across breeds, and statistical significance of the trend (^ns^*P* > 0.05; ^*^P < 0.05, ^**^P < 0.01, ^***^P < 0.001) (sd, standard deviation; pc, percentile).

	**LR**	**GR**	**GSD**	**ROTT**	**BMD**	**NEWF**
Percent scored	−0.0796%^**^	0.0948%^***^	0.0732%^**^	−0.0822*%ns*	−0.2546%^***^	−0.3996%^***^
Median score	−0.1162^***^	−0.1869^***^	−0.1069^***^	−0.0885^***^	−0.1342^***^	−0.5165^***^
Mean score	−0.2728^***^	−0.3208^***^	−0.2328^***^	−0.1519^***^	−0.2799^***^	−0.6353^***^
sd score	−0.2361^***^	−0.2439^***^	−0.2081^***^	−0.1517^***^	−0.2604^***^	−0.3418^***^
75th pc score	−0.2900^***^	−0.4762^***^	−0.3708^***^	−0.1408^***^	−0.3964^***^	−1.3148^***^

The 3 year rolling means of the median hip score of dogs born 2012–2014 were all within a narrow range: 9.00 in LR, 10.33 in GR, 11.00 in GSD, 7.33 in ROTT, 9.00 in BMD, and 10.00 in NEWF (raw data provided in Supplementary Table 1). Regression of the median hip score of animals born per year on year of birth from 1990 to 2014 yielded negative (improving) and significant (*P* < 0.001) trends/coefficients in all breeds (Table 1), ranging in magnitude from −0.0885 (ROTT) to −0.5165 (NEWF), equating to declines of −2.2 (ROTT) and −12.9 (NEWF) in median hip score over 1990–2014.

The 3 year rolling means of the mean hip score of dogs born per year 2012–2014 were 10.82 for LR, 12.85 for GR, 14.62 for GSD, 9.48 for ROTT, 11.63 for BMD, and 15.19 for NEWF. In all breeds there were more pronounced changes in the mean than for the median; reflecting the skewed distribution of hip scores. Regression of mean hip score from dogs born per year on year of birth from 1990 to 2014 showed a significant, negative (declining) trend/coefficient in all breeds (Table 1), ranging from −0.1519 in ROTT (*P* < 0.001) to −0.6353 in NEWF (*P* < 0.001), equating to declines of −3.8 (ROTT) and −15.9 (NEWF) in mean hip score over 1990–2014.

The 3 year rolling means of standard deviation (SD) of hip scores of dogs born 2012–2014 were 9.78 for LR, 9.28 for GR, 12.42 for GSD, 8.22 for ROTT, 10.34 for BMD, and 15.13 for NEWF. Regression of standard deviation of hip scores of dogs born per year on year of birth from 1990 to 2014 yielded negative, statistically significant coefficients in all breeds (Table 1), ranging from −0.1517 (*P* < 0.001) in ROTT to −0.3418 (*P* < 0.001) in NEWF, implying a reduction in variance of hip scores of dogs born per year from 1990 to 2014, equating to declines of −3.8 (ROTT) and −8.5 (NEWF) in standard deviation of hip score over 1990–2014.

The 3-year rolling means of the 75th percentile hip score of those dogs born 2012–2014 were 11.00 for LR, 13.00 for GR, 14.00 for GSD, 10.17 for ROTT, 12.25 for BMD, and 15 for NEWF Regression of the 75th percentile hip score of dogs born per year on year of birth from 1990 to 2014 showed statistically significant declining trends in all breeds (Table 1), ranging from −0.1408 in ROTT (*P* < 0.001) to −1.3148 in NEWF (*P* < 0.001), equating to declines of −3.5 (ROTT) and −32.9 (NEWF) in 75th percentile hip score over 1990–2014.

Regression coefficients for mean, standard deviation and 75th percentile of hip score were greater in magnitude than those for the median hip score (except that for standard deviation in NEWF). These parameters are affected by the skew in the distribution and so the larger declining trends compared to the median imply fewer higher scores and so a contraction of the skewed “tail” of the distribution of hip scores. Raw data on the median, mean, standard deviation and 75th percentile hip scores of registered animals born per year are given in individual breed tables in Supplementary Table 1.

#### EBV/Genetic Trend Over Year of Birth

The mean EBV for dogs of each breed born per year is shown in Table 2.

**Table 2:** Mean EBV (10 year mean = 0, standard deviation = ±20) of dogs born per year across breeds.

**YoB**	**LR**	**GR**	**GSD**	**ROTT**	**BMD**	**NEWF**
1990	30.73	33.53	20.95	23.42	21.47	26.47
1991	29.48	31.30	20.13	21.36	18.25	23.89
1992	28.06	31.37	20.30	17.80	17.99	19.31
1993	26.97	28.97	19.04	18.69	15.10	19.75
1994	25.58	26.67	19.04	17.31	13.18	19.57
1995	24.03	25.37	18.37	14.75	13.90	14.36
1996	22.07	23.50	17.43	13.70	10.94	13.94
1997	20.38	22.28	17.10	12.90	10.47	12.16
1998	18.61	19.84	15.72	13.67	12.42	12.81
1999	17.00	18.65	14.02	12.20	8.79	9.51
2000	16.91	17.93	13.10	11.79	10.89	7.72
2001	15.48	15.78	13.04	10.65	8.60	8.43
2002	14.14	14.20	12.15	10.10	8.19	5.56
2003	12.69	12.07	11.50	10.27	10.12	7.12
2004	11.50	12.14	9.61	8.55	6.94	4.80
2005	10.79	11.01	8.69	9.12	3.12	2.64
2006	8.97	9.00	7.43	7.73	6.69	4.27
2007	7.96	7.31	5.54	6.31	3.20	0.33
2008	6.83	5.43	3.96	6.41	4.13	−0.62
2009	5.08	4.30	1.81	3.63	2.00	−1.37
2010	3.62	3.07	2.50	2.99	2.86	−1.60
2011	2.95	1.81	1.78	0.59	1.71	−0.84
2012	1.24	1.26	0.89	1.59	2.10	−0.21
2013	0.84	−0.56	0.29	0.73	−0.53	1.74
2014	−0.84	−1.36	−0.95	−1.64	−1.09	0.74
2015	−1.22	−1.71	−0.57	−2.57	−1.16	1.95
2016	−2.91	−1.69	−1.32	−3.92	−2.06	1.91
2017	−4.25	−2.17	−2.63	−0.95	−3.41	0.30
2018	−5.87	−2.79	−3.79	−3.36	−1.36	−2.95

*Note that EBVs are calculated for all dogs in a pedigree, regardless of whether they have phenotypic records or not (although the accuracies of EBVS—not shown—will generally be greater for dogs with phenotypic records and/or with multiple close relative with phenotypic records)*.

All breeds show a declining trend in mean EBV for hip score of dogs born per year from 1990 to 2018 (Table 2). Regression of mean EBV on year of birth showed declining trends in all breeds: −1.2900 in LR, −1.3655 in GR, −0.9514 in GSD, −0.8894 in ROTT, −0.7732 in BMD, and −0.9038 in NEWF. All regression coefficients were statistically significantly different to zero (*P* < 0.001).

#### Score Parameters of Sires and Dams of Dogs Born Over Year of Birth

**Figure 1:**
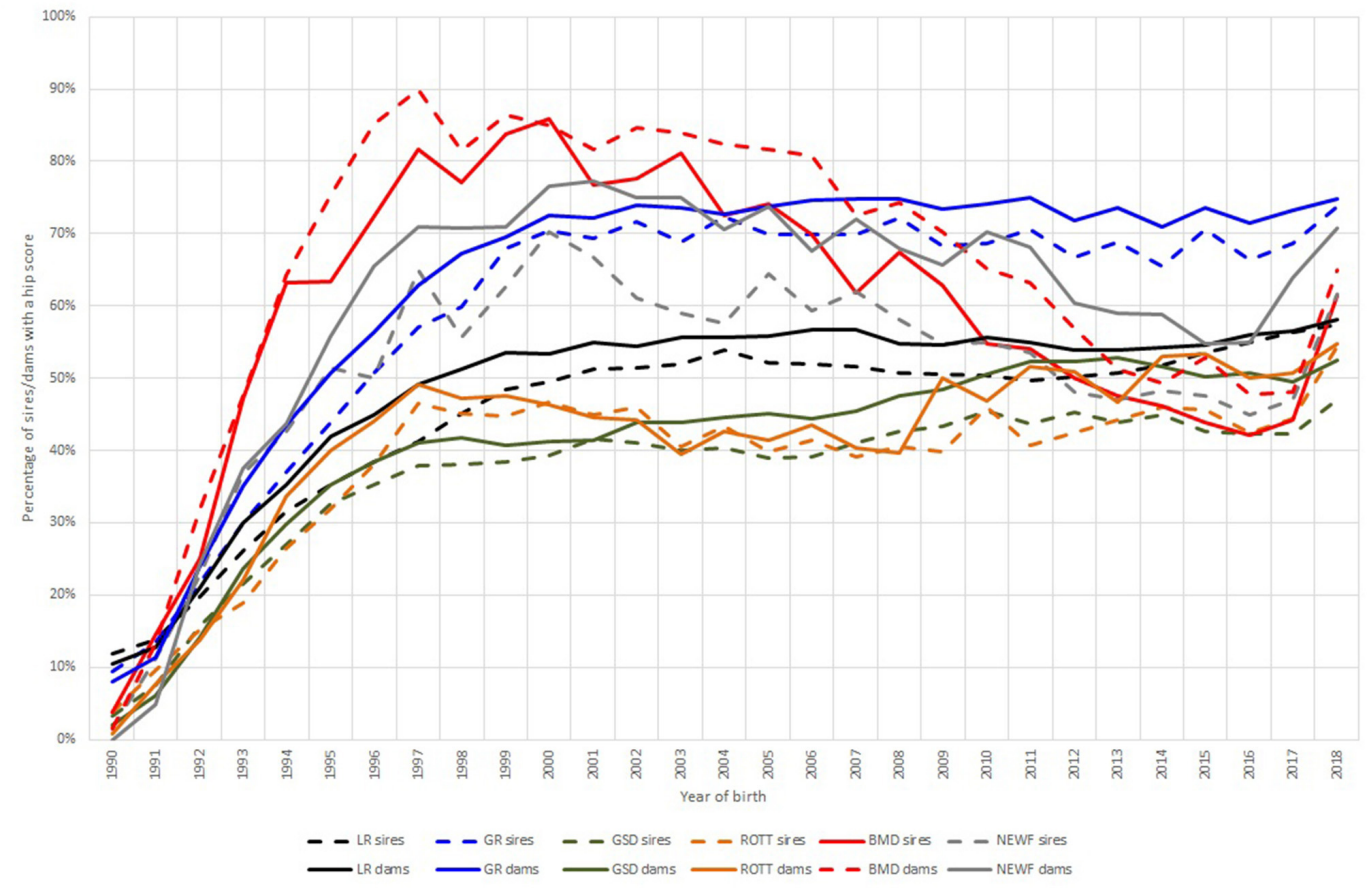
The percentage of sires (dashed lines) and dams (solid lines) (of dogs born per year) that have undergone screening and so have a hip score, in Labrador Retrievers (LR—black), Golden Retrievers (GR—blue) and German Shepherd Dogs (GSD—green), Rottweiler (ROTT—orange), Bernese Mountain Dog (BMD—red), and Newfoundland (NEWF—gray). The figure illustrates that the percentage “plateaus” in the late 1990s/early 2000s.

There was variation across breeds in the proportion of sires and dams (of dogs born per year) that have undergone hip screening, with the 3 year rolling means of the proportion of sires and dams, respectively with scores over 2016–2018 being: 56.24% and 56.93% in LR, 69.58% and 73.20% in GR, 43.93% and 50.87% in GSD, 47.06% and 51.85% in ROTT, 49.28% and 53.59% in BMD, and 51.18% and 63.19% in NEWF. Regression coefficients over year of birth of the proportion of sires and dams scored were positive (implying an increase) and statistically significant in all breeds except BMD (Table 3). However, non-linearity due to a “plateauing” of the proportion of sires and dams scored, which occurred in all breeds in the late 1990s/early 2000s (Figure 1) would have reduced the magnitude of the overall regression coefficient/trend detected, and the R-squared value, compared to a continued rate of change to that observed in earlier years. Raw data on the hip scores of sires and dams of registered dogs born per year are given in individual breed tables in Supplementary Table 2.

**Table 3:** Regression coefficients (regr.coef) (describing trend), R-squared value (R-sq) (describing proportion of variance in the dependent variable that is accounted for by the independent variable) and statistical significance of the trend (^ns^*P* > 0.05; ^*^P < 0.05, ^**^P < 0.01, ^***^P < 0.001) of the percentage of screened (i.e., with hip scores) sires and dams of dogs born per year regressed on year of birth.

	**Sires**			**Dams**		
	**Regr. coef**.	**R-sq**	**Significance**	**Regr. coef**.	**R-sq**	**Significance**
LR	1.2112	0.6681	^***^	1.1757	0.5622	^***^
GR	1.6714	0.5758	^***^	1.7251	0.5640	^***^
GSD	1.0319	0.6223	^***^	1.3277	0.7053	^***^
ROTT	0.9913	0.4951	^***^	1.1779	0.5504	^***^
BMD	0.1472	0.0038	ns	0.2715	0.0110	ns
NEWF	0.7430	0.1616	*	1.1514	0.2433	^**^

*Negative regression coefficients indicate at declining trend, and positive an increasing trend, and magnitude of ±1.00 implies a trend of increase/decrease of 1% in the proportion of that category per progressive year of birth*.

There was variation across breeds in the 3 year rolling means of hip score parameters of sires and dams of dogs born over 2016–2018 (Table 4).

**Table 4:** Three-year rolling average of hip score parameters (median, mean, standard deviation, 75th percentile of sires and dams of dogs born in 2016–2018.

		**LR**	**GR**	**GSD**	**ROTT**	**BMD**	**NEWF**
Hip score parameters of sires of 2016–2018 born dogs	Median	8.00	10.00	10.00	6.33	8.67	9.83
	Mean	8.37	10.53	10.97	7.14	9.93	10.91
	sd	5.05	4.35	5.75	3.36	6.61	5.93
	75th percentile	10.00	12.00	12.00	9.00	11.33	12.33
Hip score parameters of dams of 2016–2018 born dogs	Median	9.00	11.00	12.00	8.00	10.00	9.67
	Mean	9.32	12.18	13.85	9.58	11.18	13.09
	sd	5.52	6.09	9.24	6.73	6.07	11.03
	75th percentile	11.00	14.00	15.00	10.33	13.00	13.67

The regression coefficients/trends determined in median, mean, standard deviation, and 75th percentile of sire and dam hip score over year of birth are shown in Table 5. Regression of median hip score on year of birth determined trends that were negative (indicating decline) and statistically significant in just a few instances; ROTT sires (−0.0404, *P* < 0.01), NEWF sires and dams (−0.1089, *P* < 0.01; −0.2815, *P* < 0.001), and were positive and statistically significant in GSD dams (0.0502, *P* < 0.05, Table 5).

**Table 5:** Regression coefficients (describing trend) and statistical significance of the trend (^ns^*P* > 0.05; ^*^P < 0.05, ^**^P < 0.01, ^***^P < 0.001) of the median, mean, standard deviation and 75th percentile of hip score of sires and dams of dogs born per year regressed on year of birth.

		**LR**		**GR**		**GSD**		**ROTT**		**BMD**		**NEWF**	
Median score	Sires	−0.0079	ns	−0.0271	ns	0.0099	ns	−0.0404	^**^	−0.0182	ns	−0.1089	^**^
	Dams	−0.0133	ns	−0.0099	ns	0.0502	^*^	0.0138	ns	0.0219	ns	−0.2815	^***^
Mean score	Sires	−0.1014	^***^	−0.0930	^***^	−0.0719	^**^	−0.0560	^**^	−0.0501	ns	−0.3393	^***^
	Dams	−0.1037	^***^	−0.0660	ns	−0.0468	^***^	0.0174	ns	−0.0539	ns	−0.3354	^***^
sd score	Sires	−0.2242	^***^	−0.1913	^***^	−0.2521	^***^	−0.1510	^***^	−0.0950	ns	−0.4262	^**^
	Dams	−0.2110	^***^	−0.1595	^***^	−0.1880	^***^	0.0493	^*^	−0.1141	ns	−0.1853	^*^
75th pc score	Sires	−0.0773	^*^	−0.1025	^*^	−0.0853	^*^	−0.0520	^*^	−0.0557	ns	−0.6063	^***^
	Dams	−0.0537	ns	−0.0729	ns	−0.0390	ns	−0.0100	ns	−0.0766	ns	−0.6318	^***^

*Negative regression coefficients indicate at declining trend, and positive an increasing trend*.

A greater number of statistically significant declining trends were determined from regression of sire and dam mean hip score (Table 5), ranging from −0.0468 (GSD dams) to −0.3393 (NEWF sires).

For standard deviation of sire and dam hip score, all trends were negative and statistically significant, ranging from −0.1510 (ROTT sires) to −0.4262 (NEWF sires), with the exception of ROTT dams, which was a statistically significant positive (increasing) trend, and BMD sires and dams which were not statistically significant. For the 75th percentile of sire hip scores, all breeds had a statistically significant declining trend (ranging from −0.0520 for ROTT to −0.6063 for NEWF), except for BMD. For dams none of the breeds had a statistically significant trend in 75th percentile hip score, except for NEWF (−0.6318, Table 5). Raw data on the hip scores of sires and dams of registered dogs born per year are given in individual breed tables in Supplementary Table 2).

#### Summary of Changes in Hip Scores

A summary table showing the detection of statistically significant (*P* < 0.05), favorable (improving) trends in various hip score parameters of individuals and sires and dams over progressing year of birth is shown in Table 6. In most breeds there is some evidence of some improvement.

**Table 6:** Summary of regression coefficients of the parameters of hip score on year of birth across breeds, as described in the results.

		**LR**	**GR**	**GSD**	**ROTT**	**BMD**	**NEWF**
Individuals	% with hip score						
	Median hip score						
	Mean hip score						
	sd hip score						
	75th percentile hip score						
	EBV hip score						
Sires	% with hip score						
	Median hip score						
	Mean hip score						
	sd hip score						
	75th percentile hip score						
Dams	% with hip score						
	Median hip score						
	Mean hip score						
	sd hip score						
	75th percentile hip score						

*Where the regression coefficient was both favorable, implying improvement (for example increasing percentage with scores, or decreasing mean or standard deviation of scores) and statistically significant, it is indicated in green. When either unfavorable, or not statistically significant (or both), this is indicated in red*.

**Figure 2:**
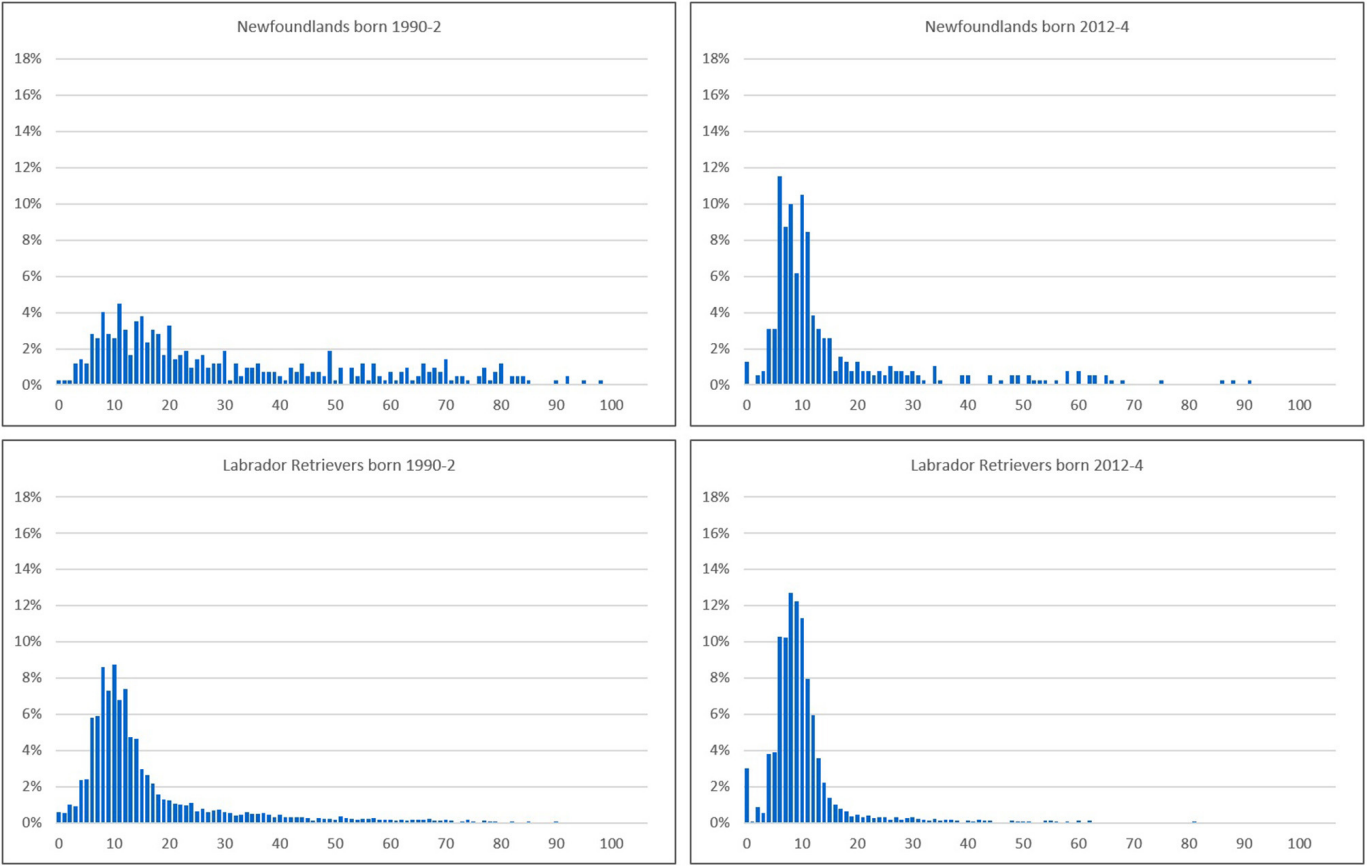
Distributions of hip scores from dogs born in 1990–2 (**Left**) and 2012–4 (**Right**) in two breeds: the NEWF (**Top**) exhibiting a major change, and the LR (**Bottom**) showing a moderate reduction in the skew/contraction in the “tail” of the distribution.

The generally larger change in parameters affected by the skewed nature of the distribution of hip score implies that improvement has taken the form of a reduction in this skew. This can be observed as a contraction in the long “tail” of high scores on the right hand side of the distribution when comparing the distribution of hip scores from 1990 to 1992 vs. 2012 to 2014 born NEWF and LR (Figure 2).

### Elbows

#### Individual Grade Parameters Over Year of Birth

There was variation across the six breeds in the proportion of registered animals born per year that had undergone screening and so had elbow grades, with the 3 year rolling mean proportion over 2012–2014 being: 5.57% in LR, 6.99% in GR, 6.35% in GSD, 7.54% in ROTT, 18.32% in BMD, and 12.26% in NEWF. Linear regression of the percentage of registered animals born that have undergone screening on individual year of birth from 1998 to 2014 determined a significant increasing trend in all breeds (Table 7). However, none of the detected regression coefficients were particularly large in magnitude.

**Table 7:** Regression coefficients (describing trend) of elbow grade parameters listed on year of birth across breeds, and statistical significance of the trend (^ns^*P* > 0.05; ^*^P < 0.05, ^**^P < 0.01, ^***^P < 0.001).

	**LR**		**GR**		**GSD**		**ROTT**		**BMD**		**NEWF**	
Proportion scored	0.3565%	^***^	0.4420%	^***^	0.4750%	^***^	0.5314%	^***^	0.1987%	^*^	0.8738%	^***^
% zero	0.3778%	^***^	0.0221%	ns	0.1723%	ns	0.7382%	ns	0.4124%	ns	−0.1472%	ns
Mean score	−0.0106	^***^	−0.0032	ns	−0.0119	^*^	−0.0242	^*^	−0.0214	^*^	−0.0094	ns
sd score	−0.0136	^**^	−0.0083	^**^	−0.0238	ns	−0.0034	ns	−0.0141	^*^	−0.0166	ns

The rolling means of the percentage of total (left + right) elbow grades that were zero in dogs born 2012–2014 were 87.29% in LR, 77.27% in GR, 81.29% in GSD, 49.07% in ROTT, 64.27% in BMD, and 69.83% in NEWF. Regression of the proportion of zero grades in dogs born per year on year of birth was only statistically significant for LR (Table 7), equating to an increase of approximately +0.4% per year.

Median total elbow grades were predominantly zero reflecting the preponderance of the zero grade, and so were not considered here.

The 3 year rolling mean of mean total elbow grade of dogs born over 2012–2014 were 0.2732 in LR, 0.4780 in GR, 0.4313 in GSD, 1.2142 in ROTT, 1.0130 in BMD, and 0.7620 in NEWF. Regression of mean total elbow grade of animals born per year on year of birth from 1998 to 2014 determined declining trends significantly different to zero in LR (−0.0106), GSD (−0.0119), ROTT (−0.0242), and BMD (−0.0214, Table 7).

For standard deviation of total elbow grade per year of birth, the 3-year rolling means over 2012–2014 per breed were: 0.8581 for LR, 1.0342 for GR, 1.0430 for GSD, 1.455 for ROTT, 1.6494 for BMD, and 1.4022 for NEWF. Regression of standard deviation of total elbow grade of animals born per year on year of birth determined that the trend was significant in only 3 breeds (−0.0136 in LR; −0.0083 GR; −0.0141 in BMD). Raw data giving the parameters of total elbow grades are given in individual breed tables in Supplementary Table 3.

#### EBV/Genetic Trend Over Year of Birth

Mean EBVs for elbow grade of dogs born per year are shown in Table 8. Regression of mean EBV of dogs born per year on year of birth were significant for all breeds except NEWF; −0.6381 (*P* < 0.001, LR), −0.0976 (*P* < 0.05, GR), −0.6828 (*P* < 0.001, GSD), −0.9283 (*P* < 0.001, ROTT), and −1.157 (*P* < 0.001, BMD).

**Table 8:** Mean elbow grade EBV (10 year mean = 0, standard deviation = ±20) of dogs born per year across breeds.

**YoB**	**LR**	**GR**	**GSD**	**ROTT**	**BMD**	**NEWF**
1998	8.64	2.40	9.00	12.28	19.65	3.57
1999	8.22	2.48	8.86	11.25	17.96	2.34
2000	7.98	1.06	8.30	10.80	16.10	2.90
2001	7.44	0.75	9.16	10.49	12.07	0.17
2002	7.43	0.65	9.06	10.51	11.71	−0.37
2003	6.54	−0.11	7.63	9.83	9.25	−0.67
2004	6.54	−0.98	6.56	9.52	11.68	1.70
2005	5.87	0.41	6.15	8.79	4.93	1.43
2006	5.08	−0.30	5.07	8.23	8.30	−1.26
2007	4.73	−0.26	4.77	8.11	8.85	−0.56
2008	4.27	0.14	3.80	7.50	5.48	−0.71
2009	3.83	−0.05	3.28	5.00	5.15	−1.68
2010	2.90	0.96	2.65	5.67	4.48	0.92
2011	2.41	−0.05	1.48	2.04	3.41	0.30
2012	0.83	1.38	0.59	3.21	1.17	−1.05
2013	0.49	0.45	0.08	0.48	1.11	−0.13
2014	−0.66	1.95	−0.95	−0.93	−0.93	−0.83
2015	−1.08	−0.13	−1.90	−3.83	−4.74	−0.27
2016	−2.40	−1.35	−2.29	−3.25	−4.55	1.59
2017	−2.92	−0.88	−2.40	−4.93	−2.73	1.33
2018	−4.48	−1.68	−3.03	−6.46	−5.07	0.89

*Note that EBVs are calculated for all dogs in a pedigree, regardless of whether they have phenotypic records or not (although the accuracies of EBVS—not shown—will generally be greater for dogs with phenotypic records and/or with multiple close relative with phenotypic records)*.

#### Grade Parameters of Sires and Dams of Dogs Born Over Year of Birth

The proportions of sires and dams of animals born per year which have an elbow grade were notably higher in BMD than other breeds in 1998–2000, with 3 year rolling mean of 22.25% and 22.45%, respectively [vs. 0.15% (GSD, dams) to 3.63% (GR, sires)]. However, by 2016–8 the disparity in the proportion of graded sires and dams across breeds had disappeared. For sires the percent graded over 2016–2018 born animals were 41.15% for LR, 48.83% for GR, 34.17% for GSD, 34.14% for ROTT, 47.80% for BMD, and 46.64% for NEWF. For dams the equivalent figures were 39.63% for LR, 45.73% for GR, 39.73% for GSD, 35.68% for ROTT, 53.12% for BMD, and 56.06% for NEWF. Regression of proportions of sires and dams with elbow grades on year of birth from 1998 to 2014 showed significant increasing trends in all breeds, except in BMD (Table 9).

**Table 9:** Regression coefficients (describing trend) and statistical significance of the trend (^ns^*P* > 0.05; ^*^P < 0.05, ^**^P < 0.01, ^***^P < 0.001) of the percentage of sires and dams of dogs born per year with elbow grades, and the percentage of sire and dam elbow grades that were zero, regressed on year of birth.

		**LR**	**GR**	**GSD**	**ROTT**	**BMD**	**NEWF**
% with grade	Sires	2.19%^***^	2.57%^***^	2.00%^***^	1.92%^***^	0.91% ns	2.70%^***^
	Dams	2.13%^***^	2.37%^***^	2.25%^***^	2.04%^***^	0.98% ns	3.06%^***^
% grade zero	Sires	0.72%^***^	0.20%*	0.39% ns	2.04%^***^	0.80%^*^	2.13%^*^
	Dams	0.48%^***^	0.77%^**^	0.70% ns	−0.83% ns	0.59% ns	−0.18% ns

*Negative regression coefficients indicate at declining trend, and positive an increasing trend, and magnitude of ±1.00 implies a trend of increase/decrease of 1% in the proportion of that category per progressive year of birth*.

The 3 year rolling means of proportion of total elbow grades that were grade zero for sires and dams of dogs born over 2016–2018 were 92.76% and 91.66% in LR, 88.17% and 81.90% in GR, 86.32% and 86.94% in GSD, 44.14% and 54.64% in ROTT, 82.69% and 61.39% in BMD, 64.21% and 76.86% in NEWF. Results of regression over year of birth of the proportion of zero grades of sires and dams are given in Table 9. For sires, significant positive trends were observed for LR (0.72% increase per year), GR (0.20% increase per year), ROTT (2.04% increase per year), BMD (0.80% increase per year) and NEWF (2.13% increase per year). For dams, significant positive trends were observed for LR (0.48% increase per year) and GR (0.77% increase per year). Raw data on the total elbow grade parameters of sires and dams of registered dogs born per year are given in individual breed tables in Supplementary Table 4).

#### Summary of Changes in Elbow Grades

A summary table showing the detection of statistically significant (*P* < 0.05), favorable (improving) trends in various elbow grade parameters of individuals and sires and dams over progressive year of birth is shown in Table 10.

**Table 10:** Summary of regression coefficients of the parameters of elbow grade on year of birth across breeds as described in the results.

		**LR**	**GR**	**GSD**	**ROTT**	**BMD**	**NEWF**
Individual	% with elbow grade						
	% zero grade						
	Mean elbow						
	sd elbow grade						
	EBV elbow grade						
Sire	% with elbow grade						
	% zero grade						
Dam	% with elbow grade						
	% zero grade						

*Where the regression coefficient was both favorable implying improvement (for example increasing percentage with grades, or decreasing mean or variance of grades) and statistically significant it is indicated in green. When either unfavorable, or not statistically significant (or both), this is indicated in red*.

## Discussion

This analysis of data from canine hip and elbow dysplasia screening schemes in the UK has demonstrated improvements in participation, phenotypic parameters and/or genetic trends for all breeds considered. Generally, greater progress was observed with respect to hip scores than elbow grades. The largest improvements in hip score data were observed in NEWF, which initially had the highest (poorest) scores. For some of the very popular breeds, for which hip dysplasia is a recognized problem (LR, GR, GSD), steady improvement was observed. In general, the changes observed in elbow grade parameters were less consistent and smaller although there were general increases detected in participation across breeds and an improving genetic trend was detected in five of the six breeds included. However, the genetic trend as determined by elbow grade EBVs was comparable with that for hip score in ROTT and exceeded it in BMD, perhaps revealing selection priorities of breeders.

The findings from this analysis of generally improving phenotypic and genetic trends are consistent with those reported in these and other breeds in a range of other countries sometimes with different evaluation schemes (12, 23, 24, 35). This indicates that selection has initiated a positive shift in assessments of hip and elbow health over time, whatever the specific details of the hip screening phenotype (phenotypes of elbow screening being more consistent). However, a recent study has reported a persisting risk of hip OA, as judged by a “distraction index” evaluation, in dogs scored as “excellent” under an “extended view” dysplasia screening scheme (36). This implies that there may be variation in hip laxity (leading to OA) which is not captured by some screening schemes, indicating that betterment of scoring parameters may be necessary to enable further improvement in reducing the ultimate risk of OA. However, selection based on EBVs has been suggested as a method with higher accuracy and so potential to induce improvements more quickly than selection upon phenotype alone, as demonstrated in previous studies (23, 25), and have profound impacts regardless of the parameters specified within a specific scheme (37).

There are several criteria which must be met to describe a screening scheme for heritable disorders as “effective,” and so several factors which may be examined to gauge the success or failure of such screening schemes. The first step that must be accomplished is a high general rate of participation, particularly among breeding individuals. This entails both a degree of acknowledgment by breeders that the condition compromises welfare and that it is present in the breed population at a heightened prevalence. Breeders must then accept the costs associated with screening as part of the regular costs of breeding. The time taken to achieve these steps may vary across different breed populations and be dependent on a number of factors, such as the severity of welfare impairment and the cost of screening (which may vary greatly, e.g., auscultation vs. an MRI scan). Participation in hip scoring, as determined in this analysis, is broadly rising for both dams and sires across most breeds in line with indicators of improvement in hip health, albeit with evidence of plateauing in recent years. Regarding the elbow scheme, five of the six breeds showed significantly rising participation of both sires and dams over time. The exception was the BMD, although it should be noted that this breed began with notably higher participation in the first instance.

The second step in determining efficacy is that, subsequent to participation, the results of screening are used in selection decisions. At first consideration, it might appear absurd that a breeder would undertake the costs of screening only then to ignore the result. However, if there is peer-pressure among contemporaries and wider society to participate in screening, then an individual may decide that being seen to participate is desirable, even if they remain skeptical of the prevalence of the condition, the severity of welfare impact or the relevance to their breeding animals. If understanding of the screening results by the public is poor and the results of screening are not publically available, then this motivation may be heightened as there is less chance of being exposed as not basing breeding decisions on the results of screening. Unfortunately, there is no feasible way of knowing to what extent phenotypic data from screening influence the breeding decisions of breeders (individually or collectively), and so parameters of the phenotypes over time must be analyzed to determine any changes and draw inferences. There were general improving trends in hip score across breeds, with evidence of changes of greater magnitude in parameters that reflect the skewed distribution of hip score, i.e., with a longer “tail” on the right hand side of the distribution (see Figure 2). For example, the regression coefficients of 75th percentile of hip score over time were between 1.6 and 3.5 times larger than those of median hip score. Greater rates of improvement in the mean, standard deviation and 75th percentile compared to the median hip score indicate fewer individuals with the high scores indicative of severe OA occurred over time. The changes appeared greatest in breeds which had the worst scores in the early 1990s (NEWF).

With regard to elbow grade the evidence of improving phenotypes was less consistent. The only breed showing steady improvement in all parameters of elbow grade (% zero grade, mean and standard deviation) was the LR, although there were significant declining trends in mean and standard deviation of elbow grade in the BMD, in mean grade in GSD and ROTT, and in standard deviation of grade in GR. The declining trends in mean elbow grade were notably larger in magnitude in ROTT and BMD than other breeds. ROTT and BMD also had markedly higher (worse) mean grades in early years, again supporting the suggestion that incidence and severity are motivators for improvement. The reported estimates of heritability for elbow score have consistently been lower than those for hip score (12, 18, 19, 25, 38), which would result in smaller genetic improvements in elbow grade than compared to hip score at the same selection intensity. Potential reasons for the lower heritability of elbow grade will include the categorical nature of the grade compared to the more continuous hip score, with each category potentially encompassing much variation in degree of pathology (particularly a grade of zero), and the plurality of individual subsets of disease included which may reduce specificity.

Improvements in the prevalence and severity of complex disease in a population, however, may come via a number of different routes, reflecting the multifactorial etiology of which genetics is just one (albeit often the single largest) contributing influence. For example, it could be that the general improvements in hip scores observed are achieved via a greater understanding of the effects of feed intake and levels of exercise in young dogs (10, 11), and subsequent appropriate changes to management. To infer selection is contributing to progress, therefore, it is necessary to examine any changes in the phenotypes of breeding animals over time, and to determine genetic changes it is necessary to examine the trend of EBVs. There were general improvements in participation in, and most parameters of, hip scoring for sires and dams across most breeds. Where the evidence of improving hip score parameters in sires and dams was weaker, despite improving trends in individual parameters and EBVs (e.g., ROTT dams, BMD), the small numbers of sires and dams with scores in early years may have had a disruptive influence in detecting trends (see Supplementary Table 2). It is possible that where participation was very low in the early years included in this study, those participating breeders may have been “early-adopters” and promoters of hip screening in these breeds, and so may also have been including some indicator of hip health in prior selection strategies. This could have introduced a bias to the data from early years, and a truer representation of the parameters may be found a few years later, when participation in screening was more the norm among sires and dams, and so the sample is more representative. While there were increases, or maintained high levels, in sire and dam participation in elbow screening across the six breeds, improvement in the proportion of which were grade zero was less consistent, possibly due to the categorical nature of grades and the preponderance of zero grades. Nevertheless, there was a significantly improving trend in proportion of at least either sire or dams with total elbow grade of zero in all breeds, except the GSD.

Trends in EBVs for hip score were favorable in all breeds, and for EBVs of elbow grade they were favorable for all breeds, except NEWF. There is no obvious reason that stands out as to why, despite an improving rate of participation of sire and dams in elbow screening (in-line with, or latterly exceeding, most of the other breeds), there were no detectable improving trends in elbow grade in this breed. The absolute numbers of sires and dams with an elbow grade was in single figures until 2006 (dams) and 2007 (sires), perhaps revealing a slow initial uptake in the breed. Under the hypothesis that initial participants in the scheme may exert a downward bias to phenotypic severity, and that gradual improved participation with the resultant use of phenotypes guiding selection, it might be expected that the elbow grades will begin to improve over an extended period of time. With regard to the remaining breeds, the generally improving genetic trend, along with a general improvement in screening participation and parameters of sires and dams implies that selection is being applied, giving rise to a consequential improvement in population-wide genetic risk.

The magnitude of the genetic response can be directly compared across breeds and phenotypes, since EBVs are centered and scaled by the mean and standard deviation in the breed over the previous decade, to give a 10 year mean EBV of zero and [genetic] standard deviation ±20. It is therefore possible to determine that, for example, the genetic progress in LR with respect to hip scores was approximately twice that for elbow grades (regression coefficients of −1.29 vs. −0.64). In these six breeds with EBVs for both hip score and elbow grade, the genetic trend was higher for hip score than elbow grade for LR, GR, GSD, and NEWF, but higher for elbow grade for ROTT and BMD, perhaps reflecting breeder objectives. The genetic trends imply that, in most breeds, selection is being applied based on the results of screening, and a genetic response elicited.

In conclusion, this study has demonstrated evidence of improving genetic trends with respect to hip score and elbow grade in six UK registered breeds in line with phenotypic improvements and participation in screening schemes. In general, improvement tends to be greater for hip score than elbow grade. This is possibly due to longstanding concerns over hip dysplasia and a more established screening scheme and culture of participation (at least in some breeds). Higher heritability estimates, due perhaps to genetic etiology but also maybe to the quantification of pathology to some degree, will also have played a role in this disparity in rates of improvement. There is variation across breeds in both the apparent prevalence of disease and the rates of improvement. Breeds with poorer hip scores or elbow grades at the outset of the periods included in this study tended to show the greatest rates of improvement.

## Data Availability Statement

All datasets generated for this study are included in the article/Supplementary Material.

## Ethics Statement

Data used in this study were measures from screening schemes to which owners voluntarily submitted their dogs. There was no research on animals, humans, or inclusion of identifiable human data.

## Author Contributions

FM and TL organized the data. HJ and TL performed the statistical analysis. All authors contributed conception and design of the study, manuscript revision, wrote sections of the manuscript, read, and approved the submitted version.

### Conflict of Interest

All authors are full-time employees of the Kennel Club.

## Supplementary Material

Supplementary Table 1 Hip score statistics of individuals born per year by breed.

Supplementary Table 2 Hip score statistics of sires and dams of dogs born per year by breed.

Supplementary Table 3 Elbow grade statistics of individuals born per year by breed.

Supplementary Table 4 Elbow grade statistics of sires and dams of dogs born per year by breed.
